# Correction to “Non‐Invasive Diagnosis of Pink Basal Cell Carcinoma: How Much Can We Rely on Dermoscopy and Reflectance Confocal Microscopy?”

**DOI:** 10.1111/srt.70308

**Published:** 2026-01-24

A. M. Witkowski, J. Łudzik, N. DeCarvalho, S. Ciardo, and C. Longo, “Non‑Invasive Diagnosis of Pink Basal Cell Carcinoma: How Much Can We Rely on Dermoscopy and Reflectance Confocal Microscopy?” *Skin Research and Technology* 22, no. 2 (2015): 230–237, https://doi.org/10.1111/srt.12254

In the published version, the second affiliation of author Joanna Łudzik was incorrect.

The correct affiliations should read:

1. Department of Dermatology, University of Modena and Reggio Emilia, Modena, Italy.

2. Department of **Bioinformatics** and Telemedicine, Jagiellonian University Medical College, Kraków, Poland.

We apologize for this error.

